# Supplementary material for: Content-rich biological network constructed by mining PubMed abstracts
Source: BMC Bioinformatics. 2004 Oct 8;5:147. doi: 10.1186/1471-2105-5-147 (PMC528731; doi:10.1186/1471-2105-5-147)
Supplement: Additional File 2 — The original results of the above study (non-essential files are deleted to keep the file size under the limit set by BMC bioinformatics). [file 1471-2105-5-147-S2.bz2 › chilibotAdditionalFile2/dip20/744ID10419942E18123/html/TOLQ_TOLR.html]

 


 **TOLQ** and **TOLR** 
  
Found 36 abstracts in PubMed, retrieved 20.  
 

 What does Google say? 
 PDF only 
| .edu only 

---

**Interactive relationship** (e.g. stimulation, inhibition, etc)

**Inhibitory relationship**- Suppressor mutant analyses have localized the  **TolQ**  TolA interaction to the first transmembrane domain of  **TolQ**  and have shown that the third transmembrane domain of  **TolQ**  interacts with the transmembrane domain of  **TolR** .  Ref: 10419942 J Bacteriol, 1999
**Neutral relationship**- Role of  **TolR**  N terminal, central, and C terminal domains in dimerization and interaction with TolA and  **tolQ** .  Ref: 10419942 J Bacteriol, 1999
- These results confirmed the essential role of the transmembrane domain of TolA in triggering interactions with  **TolQ**  and  **TolR** .  Ref: 9851983 J Bacteriol, 1998
- Analysis of sequence alignments of  **TolQ**  and  **TolR**  homologues from numerous Gram negative bacterial genomes, together with analyses of the different  **tolQ**   **tolR**  mutants, revealed that the TM domains of  **TolQ**  and  **TolR**  present structural and functional homologies not only to ExbB and ExbD of the TonB system but also with MotA and MotB of the flagellar motor.  Ref: 11722743 Mol Microbiol, 2001

**Non-interactive relationship** (e.g. studied together, co-existance, homology, etc.)

- Mutations affecting the function of TolA resulted in a lack or an alteration in TolA  **TolQ**  or  **TolR**  TolA interactions but did not affect the formation of  **TolQ**   **TolR**  complexes.  Ref: 9851983 J Bacteriol, 1998
- **TolQ** ,  **TolR** , and TolA are located in the cytoplasmic membrane where they interact with each other via their transmembrane domains.  Ref: 10633120 J Bacteriol, 2000
- However, strains with mutations in both tcpA and either  **tolQ** ,  **tolR** , or tolA were completely resistant to CTXphi infection.  Ref: 10692381 J Bacteriol, 2000
- This cluster was found to consist of seven genes in the order orf1  **tolQ**   **tolR**  tolA tolB oprL orf2.  Ref: 10735848 J Bacteriol, 2000
- Mutations in  **tolQ** ,  **tolR** , tolA, tolB, or pal genes result in sensitivity to bile salts and the leakage of periplasmic proteins.  Ref: 10633120 J Bacteriol, 2000
- In this study, we show that both  **TolQ**  and  **TolR**  proteins are essential for the TolA Pal interaction.  Ref: 11722743 Mol Microbiol, 2001
- In the present study we cloned and mutagenized the orf1,  **tolQ** ,  **tolR** , tolA, and tolB genes from P. putida KT2440, which were located upstream of the oprL gene.  Ref: 10940016 J Bacteriol, 2000
- The  **TolQ**   **TolR**  proteins energize TolA and share homologies with the flagellar motor proteins MotA MotB.  Ref: 11722743 Mol Microbiol, 2001
- Cross linking experiments have shown that the TolA transmembrane domain interacts with  **TolQ**  and  **TolR** .  Ref: 10419942 J Bacteriol, 1999
- Transmembrane domains of  **TolQ** ,  **TolR**  and TolA interact in the cytoplasmic membrane, while TolB and Pal form a complex near the outer membrane.  Ref: 9701827 Mol Microbiol, 1998
- The  **TolR**  transmembrane domain was shown to be involved in the TolA  **TolR**  and  **TolQ**   **TolR**  interactions, while  **TolR**  central and C terminal domains appeared to be involved in  **TolR**  dimerization.  Ref: 10419942 J Bacteriol, 1999
- It is organized into two complexes, one near the outer membrane between TolB and PAL and one in the cytoplasmic membrane between TolA,  **TolQ** , and  **TolR** .  Ref: 10419942 J Bacteriol, 1999
- Six of these new genes encode different homologues of the  **TolR** , TolB, and  **TolQ**  transport proteins, suggesting that adventurous motility is dependent on biopolymer transport.  Ref: 12828649 Mol Microbiol, 2003
- TolA,  **TolQ** , and  **TolR**  are inner membrane proteins, TolB is a periplasmic protein, and Pal, the peptidoglycan associated lipoprotein, is anchored to the outer membrane.  Ref: 11790745 J Bacteriol, 2002
- This change requires  **TolQ** ,  **TolR** , and a functional TolA N terminal domain.  Ref: 11418549 J Bacteriol, 2001
- Group A colicins need proteins of the Escherichia coli envelope Tol complex TolA, TolB,  **TolQ**  and  **TolR**  to reach their cellular target.  Ref: 9466263 Mol Microbiol, 1998
- Analysis of polar mutations, reverse transcriptase PCR assays, and transcriptional fusion constructs with a promoterless lacZ gene revealed that the genes are arranged in two operons orf1  **tolQ**   **tolR**  tolA tolB and oprL orf2.  Ref: 12486055 J Bacteriol, 2003
- Bacteria carrying tolA,  **tolQ**  and  **tolR**  mutations kept their colicin insensitivity as spheroplasts, just as the resistant ones.  Ref: 12094727 Folia Microbiol Praha , 2002
- Energy dependent conformational change in the TolA protein of Escherichia coli involves its N terminal domain,  **TolQ** , and  **TolR** .  Ref: 11418549 J Bacteriol, 2001
- The Tol Pal system of the Escherichia coli envelope is formed from the inner membrane  **TolQ** ,  **TolR**  and TolA proteins, the periplasmic TolB protein and the outer membrane Pal lipoprotein.  Ref: 11115123 Mol Microbiol, 2000
- The interactions of  **TolR**  with TolA and  **TolQ**  were confirmed,  **TolR**  was shown to dimerize, and the resulting dimer was shown to interact with  **TolQ** .  Ref: 10419942 J Bacteriol, 1999
- It was shown that the TolA,  **TolQ** , and  **TolR**  proteins play a critical role in the translocation of group A colicins and filamentous bacteriophage minor coat proteins g3p.  Ref: 11851406 Biochemistry, 2002
- Polar and nonpolar mutations of the P. putida  **tolQ** ,  **tolR** , tolA, and tolB genes were generated in vitro by using the omega Km r interposon, which carries two transcriptional stop signals, or a promoterless xylE cassette, lacking any transcriptional stop signal, respectively.  Ref: 10940016 J Bacteriol, 2000
- Genes homologous to  **tolQ**  and  **tolR**  proved to be very widespread being found throughout the eubacteria, and one example in the archea, this distribution argues for an ancient origin of these genes.  Ref: 11200223 J Mol Microbiol Biotechnol, 2001
- The Tol Pal system of Escherichia coli is composed of the YbgC,  **TolQ** , TolA,  **TolR** , TolB, Pal and YbgF proteins.  Ref: 11994151 Mol Microbiol, 2002
- In Pseudomonas putida, the seven genes, orf1,  **tolQ** ,  **tolR** , tolA, tolB, oprL, and orf2, which encode the proteins of this complex, are clustered in a 5.8 kb region of chromosomal DNA.  Ref: 12486055 J Bacteriol, 2003
- The  **TolQ**   **TolR**  TolA proteins form a complex in the cytoplasmic membrane, while TolB and Pal interact near the outer membrane.  Ref: 10474183 FEMS Microbiol Lett, 1999
- Transmembrane domains of  **TolQ** ,  **TolR** , and TolA interact in the cytoplasmic membrane, while TolB and Pal form a complex near the outer membrane.  Ref: 9851983 J Bacteriol, 1998
- The N terminal transmembrane domain of TolA anchors the protein to the cytoplasmic membrane and interacts with  **TolQ**  and  **TolR** .  Ref: 9851983 J Bacteriol, 1998
- Interestingly in all the cases examined the  **tolQ**  and  **tolR**  genes or their homologues appear to be present as a pair, with a potential for a tight translational regulation.  Ref: 11200223 J Mol Microbiol Biotechnol, 2001
- V. cholerae strains with insertion mutations in  **tolQ** ,  **tolR** , or tolA were reduced in their efficiency of CTXphi uptake by 4 orders of magnitude.  Ref: 10692381 J Bacteriol, 2000
- **TolQ** ,  **TolR** , and TolA inner membrane proteins of Escherichia coli are involved in maintaining the stability of the outer membrane.  Ref: 11418549 J Bacteriol, 2001
- DNA transfer requires the chromosomally encoded TolA,  **TolQ** , and  **TolR**  cytoplasmic membrane proteins.  Ref: 9537368 J Bacteriol, 1998
- This fragment was also detected in Delta  **tolQ** , Delta  **tolR** , and tolA H22P mutants but, in contrast to the parental strain, was also seen in the absence of uncouplers.  Ref: 11418549 J Bacteriol, 2001
- The predicted amino acid sequences of V. cholerae  **TolQ** ,  **TolR** , TolA, and TolB showed significant similarity to the corresponding E. coli sequences.  Ref: 10692381 J Bacteriol, 2000
